# Supplementary material for: Manual segmentation versus semi-automated segmentation for quantifying vestibular schwannoma volume on MRI
Source: Int J Comput Assist Radiol Surg. 2020 Jul 16;15(9):1445–55. doi: 10.1007/s11548-020-02222-y (PMC7419453; doi:10.1007/s11548-020-02222-y)
Supplement: Supplementary file 1 — Supplementary material 1 (pdf 801 KB) [file 11548_2020_2222_MOESM1_ESM.pdf]

For Participants

We are performing a comparative study of segmentation software for the analysis of benign brain tumours on MRI scans.

The software we will be trialling is called ImFusion. It utilises a convolutional neural network (CNN)-based machine learning approach to segment structures on medical images (MRI or CT). We will be using it to segment vestibular schwannomas on MR imaging. Vestibular schwannoma (VS) is a benign (slow growing) brain tumour of the balance nerves of the inner ear. Management algorithms for VS are based around tumour size and patient symptoms. Tumour size is generally measured in a single plane as a diameter from one margin to another. However, the tumour is a 3D structure and it is more accurate to measure its volume. In order to do this, the tumour must be segmented on MRI images. Currently, the accepted method is manual segmentation of the tumour by hand in each slice of the image. This is a time-consuming task which can be inaccurate and is subject to inter-rater variability; it is therefore seldom used clinically. We suggest that semi-automated segmentation is a quicker and easier method and is at least as accurate as manual segmentation. We hope that this study can add to the literature pool and support the use of semi-automated segmentation tools in the clinical environment.

Study Objectives

Primary – ImFusion is faster and less effortful on the part of the user for segmenting VS on MRI when compared with manual segmentation on ITK-SNAP.

Secondary – ImFusion semi-automated segmentation is as accurate as manual segmentation for vestibular schwannoma.

Study Overview

We will invite you to manually segment four different scans. These will be T1 post-contrast MRI images of patients with vestibular schwannoma. Initially we will provide a training phase, during which you will watch the study lead perform the segmentation. You will then have a trial run where the study lead will provide feedback on your segmentation technique. You may ask questions relating to segmentation technique during this time.

You will perform manual segmentation using ITK SNAP (Yushkevich et al., 2006), an open source platform for medical image viewing and segmentation. We will provide a laptop for you to use. During each segmentation, we will be timing you. You will be allowed a maximum of 10mins per scan. However, in the interests of time we recommend five minutes per scan. After you have finished the final segmentation, you will be asked several questions relating to your perception of the effort involved in the segmentation.

After this is done, you will perform semi-automated segmentation using ImFusion Labels. You will segment the same images and, as before, you will be timed. Once all four images have been segmented, you will be asked questions relating to effort.

We will then conduct a brief qualitative assessment of your experience of the segmentation.

Instructions for Participants

ITK snap (REF: Yushkevic, NeuroImage)

We will use image 203.1 for the training period.

You are allowed a maximum of 10mins for the ITK-SNAP segmentation.

The Information Below Will Be Explained By Study Lead

Open the ITK SNAP GUI using the following code: python time_itksnap.py -path_file [path_to_file] -save_file [path_to_txt_file]

The save file should be in the following format [your initials]_[scan number]_ITK

For instance:

```
python time_itksnap.py -path_file
/home/hm19/BIFSeg_Study/MRI_Data_nii/vs_gk_200.1.nii -save_file
/home/hm19/BIFSeg_Study/Participant_Data/KK/KK_200.1_ITK.txt
```

You should alter the image zoom, central point and position to suit your needs. It may be useful to have the tumor centred and as large as possible (using the zoom) for the segmentation.

1. Select polygon mode in the main toolbar (top left)
2. Pick a single ‘Active’ and ‘Paint over’ label (we suggest ‘Treatment 1’)
3. Segment the tumor in the first slice by dragging in one continuous click or by clicking at variable points around the border. Click accept once you are done in each slice.
4. Correct the segmentation for that slice using the paintbrush tool to fill in edges and small gaps.

TIP - don't try to achieve a perfect result. Segment the tumour as best you can in a reasonable time.

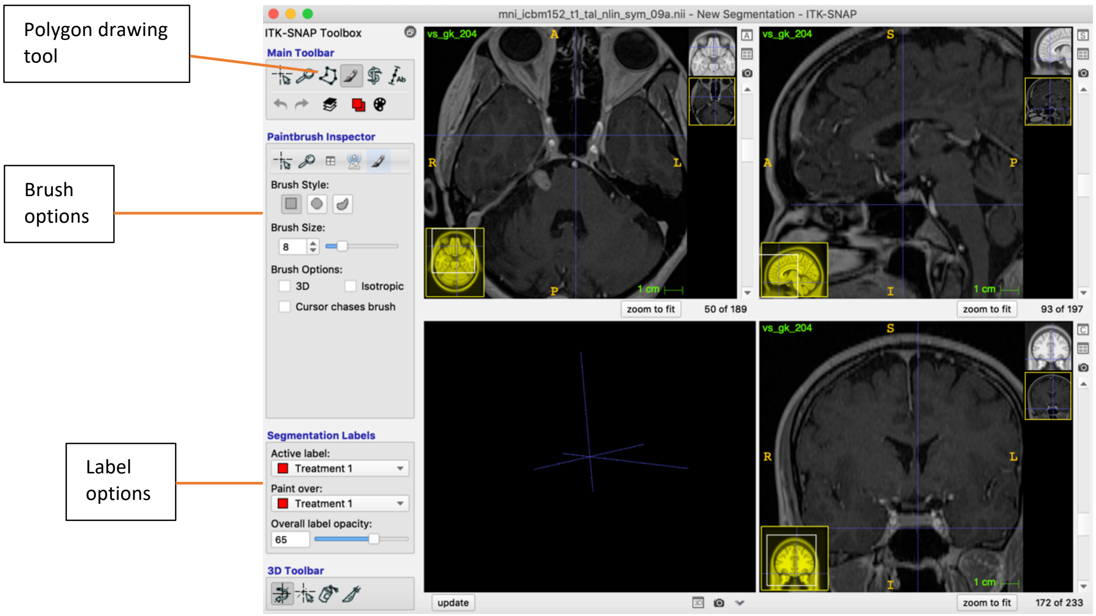

Once you are happy with the segmentation, save the file by navigating to the 'Segmentation' tab in the top left, selecting 'Save Segmentation'.
Name the file [Initials]_[Scan number]_[Program code]
e.g. HM_200.1_ITK

Close the program.

Re-open ITK-SNAP using the command line. Change the [path to file] to the next file in the series. Also change the [path to save file] to reflect the next scan.

Repeat for all the scans in the series.

After your final segmentation, you will be asked to fill in the NASA Task Load Index questionnaire relating to your experience of the software.

ImFusion

Open the ImFusion GUI by doing the following:

1. python time_imfusion.py -save_file /home/hm19/BIFSeg_Study/Participant_Data/[participant initials]/[participant initials]_[scan number]_IMF.txt
2. Alter the image zoom, central point and position to suit your needs. Here, it is useful to have the entire cranium visible.
3. Select 'Interactive Seg' tab.
4. Select 'Background label' from the list on the right side of the screen.
5. Alter the brush 'Size' and 'Adaptiveness' to suit your needs. We recommend a brush size roughly half-way along the slider, and adaptiveness set as high as is practicable.

6. Draw background labels on different structures with a range of intensities. Try to draw broad boundaries around the tumour also.
7. Now select ‘Label 1’ and draw onto the tumour, staying strictly within the tumour boundaries.

8. Inspect the resultant segmentation and accept if you are happy.
9. Perform alterations to your segmentation as needed. However:

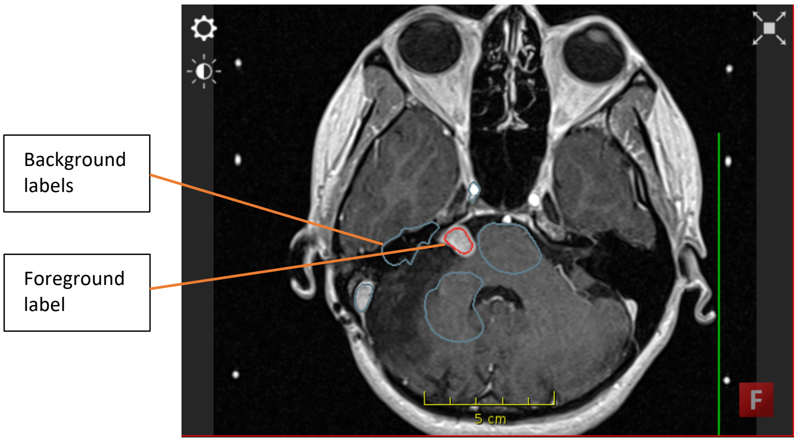

We would recommend a maximum of 2 alterations to your initial segmentation. This is to ensure optimum results.

Once you are happy with the segmentation, close the program. Your segmentation will save automatically.

Thank you for your contribution.
